# Rapid Determination of Amino Acids of *Nitraria tangutorum* Bobr. from the Qinghai-Tibet Plateau Using HPLC-FLD-MS/MS and a Highly Selective and Sensitive Pre-Column Derivatization Method

**DOI:** 10.3390/molecules24091665

**Published:** 2019-04-28

**Authors:** Wu Zhou, Yuwei Wang, Fang Yang, Qi Dong, Honglun Wang, Na Hu

**Affiliations:** 1 State Key Laboratory of Plateau Ecology and Agriculture, Qinghai University, Xining 810016, China; zhouwu870624@163.com (W.Z.); wangyuwei0507@163.com (Y.W.); yangfang2158@163.com (F.Y.); 2 College of Eco-Environmental Engineering, Qinghai University, Xining 810016, China; 3 Key Laboratory of Tibetan Medicine Research, Northwest Institute of Plateau Biology, Chinese Academy of Sciences, Xining 810008, China; qdong@nwipb.cas.cn (Q.D.); hlwang@nwipb.cas.cn (H.W.); 4 Qinghai Provincial Key Laboratory of Tibetan Medicine Research, Xining 810008, China

**Keywords:** amino acid, *Nitraria tangutorum* Bobr., Qinghai-Tibet Plateau, selective, sensitive, pre-column derivatization method, HPLC-FLD-MS/MS

## Abstract

Amino acids are indispensable components of living organisms. The high amino acid content in *Nitraria tangutorum* Bobr. fruit distinguishes it from other berry plants and is of great significance to its nutritional value. Herein, using 10-ethyl-acridine-3-sulfonyl chloride as a fluorescent pre-column labeling reagent, a method for the efficient and rapid determination of amino acid content in *N. tangutorum* by pre-column fluorescence derivatization and on-line mass spectrometry was established and further validated. The limits of detection (signal-to-noise ratio = 3) were between 0.13 and 1.13 nmol/L, with a linear coefficient greater than 0.997 and a relative standard deviation between 1.37% and 2.64%. In addition, the method required a short analysis time, separating 19 amino acids within 20 min. Subsequently, the method was used to analyze the amino acid content of *Nitraria tangutorum* Bobr. from tissues retrieved from seven regions of the Qinghai-Tibet Plateau. *Nitraria tangutorum* Bobr. was shown to contain a large amount of amino acids, with the total content and main amino acid varying between the different tissues. This research supports the nutritional evaluation, quality control, and development and utilization of *Nitraria tangutorum* Bobr.

## 1. Introduction

**Figure 1:**
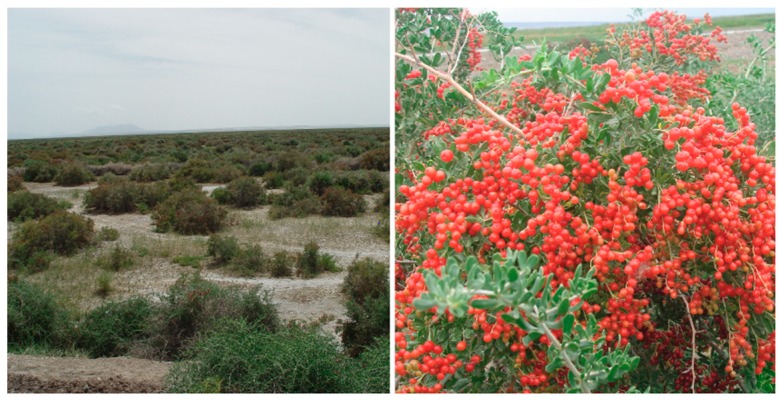
Living environment and fruits of *Nitraria tangutorum* Bobr.

*Nitraria tangutorum* Bobr. (NTB, Figure 1) belongs to the *Nitraria* genus of the Zygophyllceae, which is endemic to China [1]. It has extremely important ecological value due to its broad development prospects for the regulation of desertification and ecological environment improvement in the Qinghai-Tibet Plateau [2]. Additionally, it is relatively rich in nutrients such as amino acids, fatty acids, mineral elements, and a variety of biologically active ingredients such as polysaccharides, flavonoids, alkaloids, and anthocyanins [3]. Further, it has a broad medical and pharmacologic value, being used for the treatment of various diseases and with known antifatigue, antioxidation, hypolipidemic, and hypoglycemic properties, protection of chemical liver damage, and being involved in immune regulation [3]. Finally, given that it is a berry plant widely distributed in the Qinghai-Tibet plateau, NTB also has high edible and product development value. Previous studies have shown that the amino acid content in NTB fruit is several times that in other wild berries in the surrounding areas [4], making these berries high in nutrients.

Proteins are an essential component of cells, tissues, and organs, playing a major role in various life activities. Amino acids are the basic unit from which proteins are composed, and are therefore termed the “basic particles of life”, being involved in the metabolism of nutrients, cellular growth and development, reproduction, health, and disease. When the proportion of amino acid intake is imbalanced or lacking, abnormal physiological functions and diseases will occur [5,6]. In particular, there are eight essential amino acids that affect normal growth and development of the human body. In severe cases, a lack of these amino acids may lead to ill-health symptoms or disease [6]. The liver plays a major role in the balance of amino acids in the human body. The mismatch of the ratio of aromatic amino acids to branched chain amino acids predicts liver function damage [7,8]. Cardiovascular diseases and atherosclerosis are thought to be related to the level of sulfur-containing amino acids in the body [9]. Branched chain amino acid levels are associated with improved insulin resistance and weight loss, which are also predictors of insulin resistance in young adults [10,11]. Recently, serine, glycine [12], tryptophan [13], and branched chain amino acids [14,15] were found to be important in cancer progression. In addition, the type and content of amino acids varied in different samples or different tissues of the same sample. Therefore, it is beneficial for the identification of the authenticity of the drug to determine and analyze amino acids levels in samples [15,16]. Thus, the accurate determination of amino acids in complex biological samples or plant tissues is considered to be very important in biological, clinical, and medical fields.

Since the type and content of amino acids in complex biological samples or plant tissues is known to affect the biological, clinical, and medical applications of these materials, it is of great importance to be able to accurately determine the amino acid content of these samples for their accurate and efficient application. To date, various methods are available for the detection and analysis of amino acids. Since most amino acids show neither natural UV absorption nor fluorescence, requiring pre-column or post-column chemical derivatization to increase their detection sensitivity and improve selectivity by means of. Traditionally, the determination of amino acids has been conducted by ion-exchange chromatography followed by post-column derivatization with ninhydrin [17]. More modern analytical methods, including gas chromatography (GC) [18,19], high-performance liquid chromatography (HPLC) [20,21], and capillary electrophoresis (CE) [22,23] have been established and widely used in food, chemical, and biological sciences for amino acid analysis. Although many different types of labeling reagents such as 2,4-dinitrofluorobenzene(DNFB) [24], 9-fluorenylmethyl chloroformate (FMOC-Cl) [25], 6-aminoquinolyl-*N*-hydroxysuccinimidyl carbamate (AQC) [26], phenyl isothiocyanate (PITC) [27], o-phthaldialdehyde (OPA) [28], and 4-fluoro-7-nitro-2,1,3-benzoxadiazole (NBD-F) [29] have been developed, these have a variety of inherent shortcomings, including having short detection wavelengths, low reproducibility, time consuming and poor stability have also been reported in their applications [30]. Therefore, an improved method with high selectivity, sensitivity and reproducibility, short analysis time, and minimal sample preparation is needed and meaningful. Herein, some fluorescent labeling reagent of 2-[2-(7*H*-dibenzo[a,g]carbazol-7-yl)-ethoxy] ethyl chloroformate (DBCEC) [31], 2-(9-carbazole) ethyl chloroformate (CEOC-Cl) [32], 1,2-benzo-3,4-dihydrocarbazole-9-ethyl chloro-formate (BCEOC) [33], 10-methylacridone-2-sulfonyl chloride (MASC) [34] and 2-(11*H*-benzo[a]carbazole-11-yl) ethyl chloroformate (BCEC-Cl) [35] have been used to analyze amino acid and achieved excellent selectivity, sensitivity, reproducibility and mini-sample. In this paper, 10-ethyl-acridine-3-sulfonyl chloride (EASC) as a pre-column fluorescent labeling reagent for the determination of amino acids was established. Compared to many amino acid analysis methods, this method performed a better advantage in selectivity and sensitivity, especially in the time-saving [36].

A total of 19 amino acids were separated quickly and completely within 20 min. The method was also successfully applied in the amino acid determination of NTB from seven areas in the Qinghai-Tibet plateau. To the best of our knowledge, this is the first report of a pre-column derivatization method using HPLC-FLD-MS/MS for the determination of amino acid content in NTB. 

## 2. Results and Discussion

### 2.1. Optimization of Derivatization Conditions

To fully consider the characteristics of each type of amino acid, four amino acids, namely leucine (Leu), threonine (Thr), histidine (His), and aspartic acid (Asp), were selected as representatives to optimize the derivative process. The ratio of the derivatized reagent EASC to the total molar content of amino acids was set at 1, 2, 3, 4, 5, 6, and 7, respectively. When the ratio was less than 4, the derivatization reaction proceeded incompletely, and the fluorescence intensity was low. As the ratio increased, the fluorescence intensity also increased, reaching a maximum at a ratio of 5. There was no significant change in fluorescence intensity at higher ratios. 

Temperature was also shown to be an important factor affecting the efficiency of derivatization. Derivatization temperature was optimized within the range 10–80 °C. At temperatures below 60 °C, the derivatization reaction required long time and was incomplete. At 65 °C, the product signal intensity reached a maximum, gradually lowering upon increasing temperature to 80 °C. These observations may be due to the degradation of the derivative product or derivatized reagent at high temperatures.

The effect of derivatization time on derivatization efficiency was examined at intervals of 10 min within the range 0–40 min. The derivative product signal reached a maximum at 10 min. Unlike the derivatization of fatty acids, the derivatization of amino acids requires an alkaline environment. Therefore, the effect of different pH sodium borate buffer solutions on the derivatization efficiency was assessed. Fluorescence intensity of the derivative products was relatively weak at pH values lower than 8.0 or higher than 9.5. Between pH 8.0 and 9.5, the fluorescence intensity of the derivative product was strong, reaching a maximum at pH 9.0. 

Thus, the optimal derivatization conditions were selected as a sodium borate buffer solution at pH 9.0, derivatization reagent equivalent to 5 times of total number molar of amino acids, in a water bath at 65 °C for 10 min. Under this condition, the reaction could be ensured to react completely.

### 2.2. Chromatographic Separation and Mass Spectrometry Identification

#### 2.2.1. HPLC Separation

In order to obtain better chromatographic separation conditions, the effects of the mobile phase and the choice of chromatographic column on the separation results were examined. When methanol and acetonitrile were used as the mobile phase, the latter achieved fast separation with a sharp chromatographic peak. Spherisorb C18 (200 mm × 4.6 mm, 5 µm), Akasil-C18 column (200 mm × 4.6 mm, 5 μm), Hypersil C18 (200 mm × 4.6 mm, 5 µm), Hypersil BDS C18 (200 mm × 4.6 mm, 5 µm), and Hypersil BDS C8 (200 mm × 4.6 mm, 5 µm) columns were used to separate 19 amino acids. The results showed that C18 columns was better than the C8 columns. Although the Hypersil BDS C18 column could also be used for amino acid separation, the process took approximately 60 min. The Akasil-C18 column could achieve rapid and complete separation of 19 amino acids within 20 min.

#### 2.2.2. MS Identification

**Figure 2:**
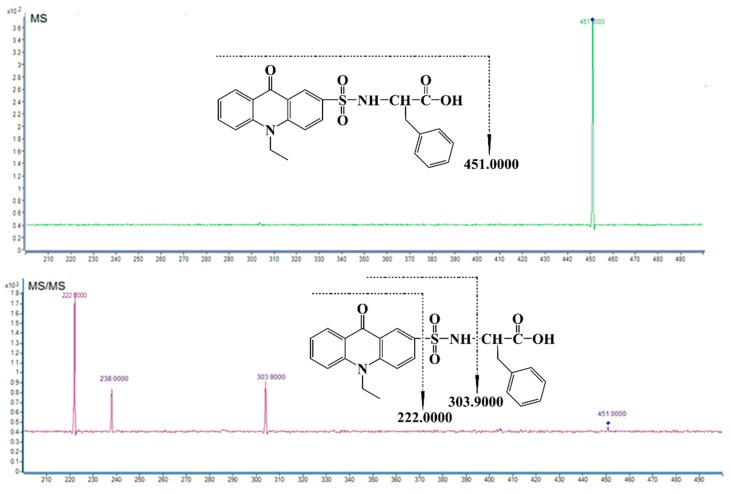
MS spectra of the representative phenylalanine derivative and fragmentation pattern of the protonated molecular ion.

The amino acid derivatives were further identified by electrospray ionization in the positive ion mode. The molecular ion peaks and fragment ion peaks produced by the amino acid derivatives are listed in Table 1. The mass spectrum of the l-phenylalanine acid derivative as the representative is shown in Figure 2. The molecular ion peak at *m/z* 451.0 had a high specific intensity; *m/z* 222.0, 238.0, and 303.9 are the fragment ion peaks generated after molecular ion collisions. The fragment ion peak at *m/z* 222.0 was derived from cleavage of the C-SO_2_ bond of the amino acid derivative. The fragment at *m/z* 303.9 was derived from cleavage of the N-C bond of the internal structure for the N-side chain. Therefore, fragment ion peaks *m/z* 222.0 and *m/z* 303.9 are valid markers to identify amino acid derivatives. This cleavage mode is consistent with that reported in the literature [37,38,39].

### 2.3. Method Valuation

The linearity, limits of detection, limits of quantitation, precision, and accuracy of the established method were also verified according to the methods reported in the literature (Table 1). In the 2.0–800.0 nmol/L range, the response value was linearly regressed to the amino acid concentration, and the linear correlation coefficient was greater than 0.997. Limits of detection are defined as the concentration at which a signal-to-noise ratio of 3 is produced, in this case within the range 0.13–1.13 nmol/L. The limits of quantitation are defined as the concentration at which a signal-to-noise ratio of 10 is produced, herein between 0.38 nmol/L and 3.10 nmol/L. The precision was obtained by analyzing the actual sample in parallel three times. The results showed that the precision of the method (expressed by the relative standard deviation) was between 1.37% and 2.64%. To evaluate the accuracy of the method, recovery experiments were performed by spiking blank samples with the amino acid standard mixture at three different concentrations in three replicates. The NTB sample without the standard added was used as the blank sample. The spiked sample went through the entire experimental process, including acid hydrolysis, derivation, and injection analysis. The formula ‘(measured value − background value)/added value * 100%’ was used to calculate the recoveries. The recovery of all amino acids was determined to be between 93.2% and 103.4%. 

### 2.4. Analysis and Evaluation of Amino Acids in Nitraria tangutorum Bobr

**Figure 3:**
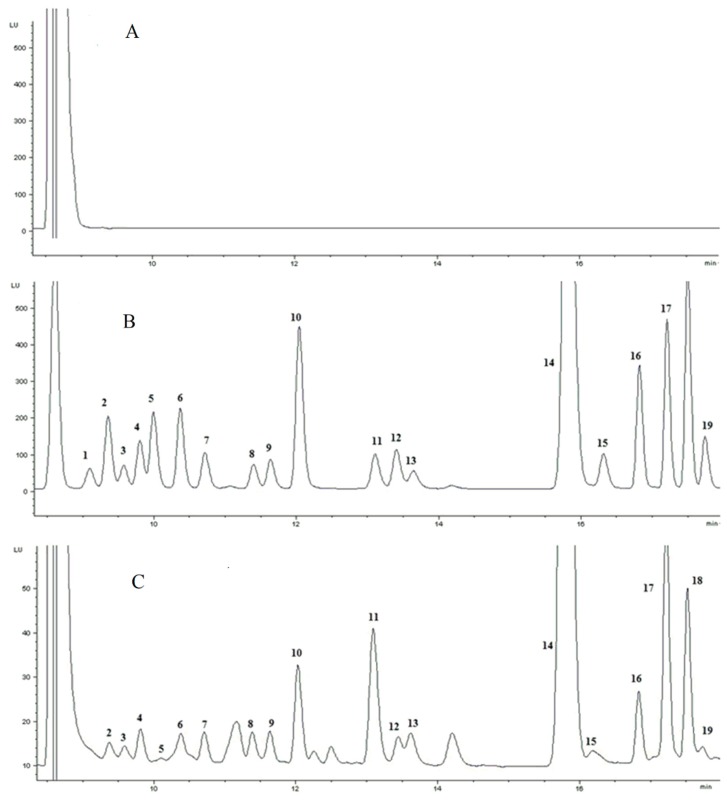
Representative chromatograms for blank (**A**), standards (**B**) and the typical chromatograms for the amino acids derivatives in peel and pulp of *Nitraria tangutorum* Bobr. (**C**) peak labels: 1. cysteine HCl (Cys), 2. Histidine (His), 3. ornithine (Orn), 4. arginine (Arg), 5. lysine (Lys), 6. Serine (Ser), 7. aspartic acid (Asp), 8. glutamic acid (Glu), 9. threonine (Thr), 10. Glycine (Gly),11. tryptophan (Trp), 12. alanine (Ala), 13. tyrosine (Tyr), 14. proline (Pro), 15. methionine (Met), 16. valine (Val), 17. phenylalanine (Phe), 18 isoleucine (Ile and 19. leucine (Leu).

The above established method was used to analyze the amino acid content in various tissues of NTB. The analyzed amino acids included free amino acids and the amino acids disassociated from the protein/polypeptide. An excess of derivatized reagent EASC was used to ensure that the amino acids in the sample were fully derivatized. In order to ensure the accuracy of the results, a standard sample was injected every five injections to correct any error which could occur in the retention time. The chromatograms of the blank, amino acid derivative in standard sample, and NTB peel and pulp samples are shown in Figure 3. The detailed data of amino acid content measured in NTB peel and pulp, seeds, and leaves are provided in Table 2, Table 3 and Table 4, respectively. All NTB tissues analyzed contained a large number of amino acids, with the total amino acid content being largest in the peel and pulp sample (110.04–122.41 mg/g), followed by leaves (91.99–98.29 mg/g), and seeds (69.98–77.20 mg/g). In addition, the main amino acids varied among the different tissues. The analysis of amino acids in NTB peel and pulp showed that the main amino acid was proline (Pro), with its content reaching between 26.08 and 30.13 mg/g, accounting for more than 20% of the total amino acid content. This is related to the habitat in which it is located. Pro is a major component of plant proteins, being used as a cytoplasmic osmo-regulatory substance and playing an important role in stabilizing the structure of biological macromolecules, reducing cell acidity, relieving ammonia toxicity, and regulating the cell redox potential as an energy pool [40]. Under adverse conditions (dry, saline, hot, cold, frozen), the Pro content in plants increases significantly, and reflects plant resistance, with plants with strong drought resistance often accumulating Pro [41]. In addition, the alanine (17.98–21.22 mg/g), Thr (16.79–19.76 mg/g), phenylalanine (8.09–11.22 mg/g), and Leu (5.63–9.08 mg/g) contents in NTB peel and pulp were higher than that of other amino acids. The main amino acids in NTB seeds were glutamic acid (Glu) and arginine (Arg), with their content being between 14.79–17.87 mg/g and 8.78–13.16 mg/g, respectively. Glu is a non-essential amino acid under normal conditions. However, its demand exceeds the body’s ability to synthesize it under harsh external stress conditions, because its content is also related to its habitat. Arg is considered a conditional non-essential amino acid. Studies have shown that Arg has insulin-promoting and secretory effects, promoting growth and wound healing. Supplementation of Arg at high temperatures could reduce protein catabolism during heat stress [42]. In addition, the Asp (6.32–8.09 mg/g) and Leu (4.79–6.04 mg/g) content in NTB seeds was also higher than that of other amino acids. Pro was the main amino acid in NTB leaves, with a content between 17.87 and 20.12 mg/g, related to the plant’s resistance. Glu and Asp, with a content of 16.87–18.93 mg/g and 7.98–10.34 mg/g, respectively, were also present. Asp is ubiquitous in biosynthesis, being a synthetic precursor of some essential amino acids in the organism such as lysine, Thr, isoleucine, methionine, and purine and pyrimidine bases, and being shown to improve myocardial contractile function, enhance liver function, and eliminate fatigue [42].

NTB was rich in amino acids of the seven different regions in the Qinghai-Tibet plateau. The types and total amount of amino acids in NTB varied according to the region from which the plants were retrieved. The ratio of the eight essential amino acids contained in NTB peel and pulp from the seven regions was generally high. The Food and Agriculture Organization and the World Health Organization (FAO/WHO) proposed that the ideal protein should meet the following conditions: the ratio of total content of essential amino acids (E) to total content of amino acids (T) (E/T) value should be above 40%, and the E to total content of non-essential amino acids (NE) (E/NE) value should be above 60%. The E/T value of NTB peel and pulp from six regions exceeded the FAO/WHO standard value of 40%. Only the E/T value (39.4%) of NTB peel and pulp from Delingha was close to 40%. The E/NE values of NTB peel and pulp from all regions were all greater than the FAO/WHO standard value 60%. However, the E/T values of NTB seeds and leaves were all lower than the FAO/WHO standard value of 40%. Moreover, the E/NE values of NTB seeds and leaves were all lower than the FAO/WHO standard value of 60%. 

Therefore, NTB fruit peel and pulp contained eight essential amino acids in high amounts, thus being of a high nutritional value. Based on this, a variety of foods rich in amino acids based on NTB fruit can be developed. Although the E/T and E/NE values of NTB seeds and leaves were lower than the standard value, the main amino acid was significant. The seeds and leaves could be used as an extraction source of major amino acids for medicinal products, the development of anti-fatigue products, or as bio-fertilizers. From the above amino acid analysis in NTB, it can be seen that NTB fruit is rich in amino acids with high nutritional and health value. Thus, NTB could be used as a basic raw material for food and healthcare products.

## 3. Materials and Methods

### 3.1. Instruments

The HPLC analysis system was an Agilent 1100 series HPLC (Agilent Technologies Co., Palo Alto, CA, USA) equipped with a quaternary pump (model G1311A), an online-degasser (model G1322A), a thermostated column compartment (model G1316B), an autosampler (model G1329A), and a FLD detector (model G1321A). The mass spectrometer (MSD Trap SL,) from Bruker Daltonik, (Bremen, Germany) was equipped with an electrospray ionization (ESI) source (model G1948A). The HPLC-MSD system was controlled by the Agilent Chemstation software (version B.01.01). Derivatives were separated on an Akasil-C18 column (200 × 4.6 mm, 5 µm i.d. (Agilent Technologies Co., Ltd.).

### 3.2. Reagents and Chemicals

**Scheme 1:**
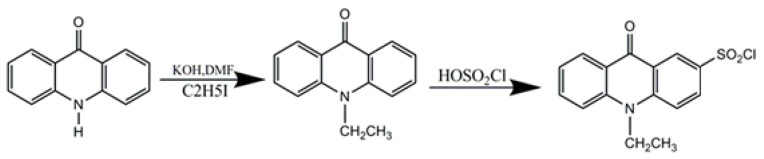
Synthesis process of the derivatized reagent EASC.

Amino acid standards of proline (Pro), cysteine HCl (Cys), valine (Val), methionine (Met), phenylalanine (Phe), histidine (His), ornithine (Orn), arginine (Arg), lysine (Lys), serine (Ser), aspartic acid (Asp), glutamic acid (Glu), glycine (Gly), threonine (Thr), tryptophan (Trp), alanine (Ala), tyrosine (Tyr), leucine (Leu), and isoleucine (Ile) were purchased from Sigma Chemical Co. (St. Louis, MO, USA). The derivatization reagent EASC was synthesized in our laboratory as previously published [40] (Scheme 1). HPLC grade acetonitrile and methanol were obtained from Tianjin Kemiou Chemical Reagent Company, Tianjin, China. Water was purified on a Milli-Q system (Millipore, Bedford, MA, USA). All other reagents used were also of analytical grade unless otherwise stated.

### 3.3. Preparation of Plant Material

Mature fruits and leaves of NTB were collected from seven regions in the Qinghai-Tibet plateau in October 2018 and were identified by senior engineer Changfan Zhou. Detailed sample information is listed in Table 5. The collected samples were dried naturally, and the fruits were divided into two parts consisting of peel and pulp or seeds. All dried samples were smashed and sieved through a 60 mesh sieve. Subsequently, a 20 mg sample was weighed and dissolved in 1.1 mL of HCl solution at a concentration of 6 mol/L. The sample was then placed in an oven at 110 °C for 24 h for hydrolysis of amino acid chains such as proteins and peptides into single amino acids. Finally, 153 μL of 1 mol/L NaOH was added into 200 μL of the hydrolyzed HCl sample solution and a 200 μL sample was derivatized.

### 3.4. Preparation of Solutions

EASC (4.85 mg) was weighed and dissolved in 15 mL of acetonitrile solution, producing a derivatized reagent solution of 1 × 10^−3^ mol/L. Each amino acid standard was weighed and a stock solution was prepared at a concentration of 5 × 10^−3^ mol/L. Poorly soluble amino acids were solubilized by adding a few drops of HCl solution. A 1 × 10^−4^ mol/L mixed working solution of amino acids was obtained by adding each stock solution and dilution. The above derivatization reagent and standard solutions were stored at –4 °C when not in use.

### 3.5. Derivatization Procedure

**Scheme 2:**
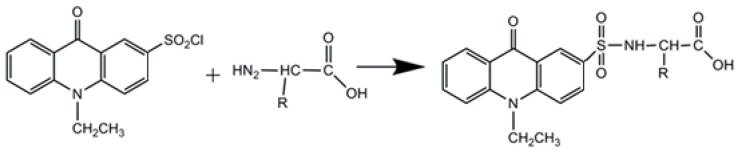
Derivatization reaction process of the derivatized reagent EASC and amino acid.

The amino acid standard solution (20 μL) or 200 μL of the sample solution, 110 μL of the derivatization reagent, 100 μL of borax buffer solution at a concentration of 0.1 mol/L (pH 9.0), and 80 μL of acetonitrile solution were added into a 2 mL vial. The vial was then sealed and placed in a water bath at 65 °C for 10 min. Subsequently, the mixed solution was cooled to room temperature and passed through a 0.22 μm nylon membrane. A 10 μL aliquot was taken for further HPLC analysis. The derivatization reaction process of the derivatized reagent EASC and amino acids is shown in Scheme 2.

### 3.6. HPLC Separation and MS Condition

The fluorescence excitation and emission wavelength were set at 262 nm and 425 nm. The A and B mobile phases were 5% acetonitrile plus 95% water and 100% acetonitrile, respectively. The gradient condition was set as follows: 0 min = 0% B, 1 min = 15% B, 4 min = 24% B, 5 min = 29% B, 7 min = 33% B, 9 min = 38% B, 10 min = 47% B, 12 min = 51% B, 13 min = 54% B, 15 min = 80% B, 18 min = 100% B, and 20 min = 100% B. The flow speed was 1.0 mL/min and the injection volume 10 μL. The column temperature was set at 35 °C. It should be balanced with initial concentration for 5 min before each injection. Chromatographic peaks were characterized by retention times and simultaneously identified by on-line MS. MS conditions were ionized electrospray ionization source in the positive ion mode, spray pressure 60 psi, a dry gas flow rate of 9 L/min, dry gas temperature at 350 °C, Vap temperature at 450 °C, and a capillary voltage of 3500 V.

## 4. Conclusions

Based on the derivation reagent EASC, a method for the analysis of amino acid content was established. At the same time, the derivatization and chromatography separation conditions were optimized. The derivatized product after the reaction was simultaneously monitored by MS, and the method was further verified by linearity, sensitivity, accuracy, and precision. The method showed a high sensitivity and good stability, and was subsequently successfully applied to analyze the amino acid content in different tissues of NTB obtained from seven different regions. The results showed that the different tissues all contained a large amount of amino acids, with the total amount in NTB peel and pulp being the highest, followed by leaves. Further, the total amount of amino acids in NTB seeds was relatively low. In addition, the main amino acid varied according to the different tissues, being Val and tyrosine in NTB peel and pulp, Glu and Arg in NTB seeds, and Val, Glu, and Asp in NTB leaves. There was no difference in the type of amino acid in the same tissues from different regions, but there existed some differences in content. From the evaluation of amino acids in NTB samples of different tissues, it was concluded that NTB has a high nutritional value and could be used for the development of various products. 

## Figures and Tables

**Table 1:** Molecular ions [M + H]^+^, retention time, correlation coefficients, limits of detection, limits of quantification, recovery, and precision values of amino acid derivatives.

Amino Acid	MS [M + H]^+^	Retention Time (min)	Correlation Coefficient	LODs (nmol/L)	LOQs (nmol/L)	Recovery (%)	RSD (%)
Cys	461.5	9.094	0.9981	0.60	1.45	96.4	1.56
His	441.1	9.352	0.9978	0.89	2.52	96.9	1.73
Orn	418.1	9.571	0.9992	0.22	0.58	94.5	1.65
Arg	460.1	9.798	0.9989	0.19	0.63	103.4	2.12
Lys	432.1	9.989	0.9991	1.13	3.10	98.7	2.32
Ser	391	10.355	0.9995	0.45	1.13	95.8	1.76
Asp	417.9	10.713	0.9983	0.18	0.49	97.6	2.21
Glu	433	11.394	0.9985	0.29	0.67	99.1	2.09
Thr	405	11.633	0.9987	0.15	0.53	95.4	1.87
Gly	361	12.036	0.9993	0.14	0.38	93.8	2.14
Trp	490	13.102	0.9978	0.38	1.09	98.0	2.05
Ala	375	13.403	0.9996	1.06	2.97	94.7	2.64
Tyr	466.9	13.641	0.9991	0.31	0.89	98.1	1.89
Pro	401	15.812	0.9972	0.27	0.92	101.3	1.57
Met	435.1	16.311	0.9988	0.14	0.49	95.6	1.37
Val	403	16.817	0.9979	0.23	0.58	96.9	2.27
Phe	451.1	17.205	0.9991	0.78	2.21	98.5	2.51
Ile	417.1	17.497	0.9984	0.16	0.42	95.2	1.44
Leu	417.1	17.738	0.9993	0.13	0.40	99.9	2.23

**Table 2:** The measured contents of amino acids in peel and pulp of *Nitraria tangutorum* Bobr. (mg/g).

Amino Acid	Region						
	Dagele	Zongjia	Nuomuhong	Delingha	Hedong	Dulan	Gahai
His	1.35 ± 0.04	1.76 ± 0.07	2.12 ± 0.10	1.89 ± 0.08	1.65 ± 0.06	2.03 ± 0.09	1.78 ± 0.08
Orn	0.42 ± 0.02	0.35 ± 0.01	0.47 ± 0.02	0.33 ± 0.01	0.27 ± 0.01	0.65 ± 0.03	0.29 ± 0.02
Arg	2.87 ± 0.11	3.12 ± 0.13	3.34 ± 0.11	2.99 ± 0.10	3.54 ± 0.12	4.13 ± 0.14	3.77 ± 0.13
Lys *	0.24 ± 0.01	0.18 ± 0.02	0.43 ± 0.02	0.11 ± 0.01	0.23 ± 0.01	0.31 ± 0.01	0.27 ± 0.01
Ser	2.10 ± 0.09	2.97 ± 0.12	3.54 ± 0.15	4.11 ± 0.14	3.06 ± 0.11	2.96 ± 0.09	4.03 ± 0.16
Asp	1.51 ± 0.04	1.76 ± 0.04	1.32 ± 0.05	1.65 ± 0.04	1.87 ± 0.06	2.02 ± 0.07	1.43 ± 0.05
Glu	0.98 ± 0.03	0.79 ± 0.02	1.11 ± 0.04	0.87 ± 0.02	1.54 ± 0.03	1.05 ± 0.03	0.95 ± 0.02
Thr *	17.21 ± 0.64	18.41 ± 0.67	19.76 ± 0.69	16.79 ± 0.58	19.23 ± 0.66	18.56 ± 0.54	18.09 ± 0.63
Gly	1.09 ± 0.02	1.32 ± 0.03	0.98 ± 0.03	1.43 ± 0.04	1.25 ± 0.02	0.87 ± 0.02	1.29 ± 0.05
Trp *	1.44 ± 0.04	2.12 ± 0.05	2.34 ± 0.03	4.33 ± 0.12	3.05 ± 0.06	1.75 ± 0.05	1.54 ± 0.04
Ala	19.81 ± 0.72	17.98 ± 0.65	19.04 ± 0.70	18.90 ± 0.66	19.03 ± 0.71	17.98 ± 0.69	21.22 ± 0.81
Tyr	8.05 ± 0.31	8.97 ± 0.34	9.87 ± 0.37	10.11 ± 0.41	9.07 ± 0.33	9.98 ± 0.38	8.79 ± 0.32
Pro	27.47 ± 1.08	28.07 ± 1.02	29.54 ± 1.21	28.05 ± 1.14	30.13 ± 1.20	28.09 ± 1.07	26.08 ± 0.99
Met *	0.43 ± 0.02	0.35 ± 0.01	0.61 ± 0.02	0.33 ± 0.01	0.67 ± 0.02	0.54 ± 0.02	0.89 ± 0.03
Val *	5.12 ± 0.21	6.02 ± 0.19	6.78 ± 0.25	7.03 ± 0.26	6.92 ± 0.25	4.89 ± 0.17	7.43 ± 0.25
Phe *	9.14 ± 0.31	10.11 ± 0.38	11.22 ± 0.40	8.09 ± 0.30	8.96 ± 0.29	10.22 ± 0.40	9.99 ± 0.39
Ile *	3.12 ± 0.12	4.05 ± 0.11	4.32 ± 0.14	2.98 ± 0.09	3.05 ± 0.10	4.87 ± 0.15	4.77 ± 0.13
Leu *	7.69 ± 0.29	5.63 ± 0.21	8.05 ± 0.31	6.09 ± 0.23	7.24 ± 0.26	7.06 ± 0.27	9.08 ± 0.33
T	110.04 ± 1.57	113.96 ± 1.52	124.84 ± 1.72	116.08 ± 1.59	120.76 ± 1.66	117.96 ± 1.55	121.69 ± 1.59
E	44.39 ± 0.81	46.87 ± 0.83	53.51 ± 0.90	45.75 ± 0.75	49.35 ± 0.81	48.2 ± 0.76	52.06 ± 0.86
NE	65.65 ± 1.34	67.09 ± 1.27	71.33 ± 1.46	70.33 ± 1.39	71.41 ± 1.45	69.76 ± 1.34	69.63 ± 1.34
E/T (%)	40.34	41.13	42.86	39.41	40.87	40.86	42.78
E/NE (%)	67.62	69.86	75.02	65.05	69.11	69.09	74.77

Note: *: essential amino acids; E: total content of essential amino acids; NE: total content of non-essential amino acids; T: total content of amino acids.

**Table 3:** The measured contents of amino acids in seeds of *Nitraria tangutorum* Bobr. (mg/g).

Amino Acid	Region						
	Dagele	Zongjia	Nuomuhong	Delingha	Hedong	Dulan	Gahai
His	1.54 ± 0.06	1.35 ± 0.04	1.62 ± 0.05	2.13 ± 0.07	1.98 ± 0.06	1.80 ± 0.04	1.47 ± 0.05
Orn	0.58 ± 0.02	0.34 ± 0.01	0.65 ± 0.02	0.29 ± 0.01	0.51 ± 0.01	0.49 ± 0.01	0.36 ± 0.01
Arg	10.98 ± 0.38	12.06 ± 0.41	13.16 ± 0.45	10.78 ± 0.42	8.93 ± 0.29	9.56 ± 0.39	8.78 ± 0.31
Lys *	0.43 ± 0.01	0.34 ± 0.01	0.29 ± 0.01	0.62 ± 0.02	0.52 ± 0.02	0.45 ± 0.01	0.69 ± 0.02
Ser	3.90 ± 0.14	4.31 ± 0.16	3.89 ± 0.11	5.02 ± 0.19	7.08 ± 0.25	2.97 ± 0.11	2.76 ± 0.11
Asp	6.91 ± 0.24	6.56 ± 0.26	5.98 ± 0.23	8.09 ± 0.31	6.32 ± 0.22	6.70 ± 0.25	6.99 ± 0.28
Glu	16.21 ± 0.61	14.89 ± 0.53	17.34 ± 0.60	17.87 ± 0.63	16.43 ± 0.61	15.89 ± 0.59	14.79 ± 0.55
Thr *	2.22 ± 0.07	2.67 ± 0.08	1.99 ± 0.07	3.13 ± 0.11	2.78 ± 0.09	4.01 ± 0.13	3.90 ± 0.14
Gly	4.31 ± 0.16	5.12 ± 0.20	4.67 ± 0.18	3.32 ± 0.13	6.03 ± 0.24	5.21 ± 0.21	3.65 ± 0.13
Trp *	0.98 ± 0.03	1.23 ± 0.04	0.87 ± 0.03	0.62 ± 0.02	1.15 ± 0.03	0.96 ± 0.03	0.69 ± 0.02
Ala	3.65 ± 0.13	2.87 ± 0.08	3.54 ± 0.12	4.12 ± 0.16	2.98 ± 0.12	3.76 ± 0.13	3.02 ± 0.12
Tyr	1.76 ± 0.07	1.23 ± 0.03	2.03 ± 0.07	1.69 ± 0.06	0.98 ± 0.03	1.54 ± 0.05	1.43 ± 0.04
Pro	4.13 ± 0.15	5.09 ± 0.20	3.89 ± 0.14	4.54 ± 0.17	3.67 ± 0.15	5.54 ± 0.22	3.67 ± 0.13
Met *	0.23 ± 0.01	0.11 ± 0.01	0.34 ± 0.01	0.24 ± 0.01	0.17 ± 0.01	0.09 ± 0.01	0.14 ± 0.01
Val *	4.33 ± 0.15	4.78 ± 0.18	3.98 ± 0.16	3.34 ± 0.13	5.12 ± 0.19	4.68 ± 0.17	5.42 ± 0.21
Phe *	3.23 ± 0.14	3.45 ± 0.12	3.76 ± 0.13	2.87 ± 0.09	4.05 ± 0.12	4.65 ± 0.18	3.97 ± 0.16
Ile *	2.35 ± 0.09	1.89 ± 0.07	3.11 ± 0.12	2.67 ± 0.09	2.09 ± 0.08	3.56 ± 0.13	2.54 ± 0.10
Leu *	5.46 ± 0.21	5.78 ± 0.24	6.04 ± 0.23	4.79 ± 0.18	5.88 ± 0.23	5.34 ± 0.20	5.41 ± 0.24
T	73.2 ± 0.88	74.07 ± 0.87	77.15 ± 0.90	76.13 ± 0.93	76.67 ± 0.89	77.20 ± 0.91	69.68 ± 0.84
E	19.23 ± 0.32	20.25 ± 0.34	20.38 ± 0.34	18.28 ± 0.28	21.76 ± 0.35	23.74 ± 0.37	22.76 ± 0.40
NE	53.97 ± 0.82	53.82 ± 0.79	56.77 ± 0.84	57.85 ± 0.89	54.91 ± 0.82	53.46 ± 0.83	46.92 ± 0.74
E/T (%)	26.27	27.34	26.42	24.01	28.38	30.75	32.66
E/NE (%)	35.63	37.63	35.90	31.60	39.63	44.41	48.51

Note *: essential amino acids; E: total content of essential amino acids; NE: total content of non-essential amino acids; T: total content of amino acids.

**Table 4:** The measured contents of amino acids in leaves of *Nitraria tangutorum* Bobr. (mg/g).

Amino Acid	Region						
	Dagele	Zongjia	Nuomuhong	Delingha	Hedong	Dulan	Gahai
His	1.34 ± 0.03	1.54 ± 0.05	2.11 ± 0.08	2.09 ± 0.07	1.87 ± 0.06	1.02 ± 0.03	2.05 ± 0.05
Orn	0.23 ± 0.01	0.11 ± 0.01	0.32 ± 0.01	0.09 ± 0.01	0.33 ± 0.01	0.26 ± 0.01	0.29 ± 0.01
Arg	3.87 ± 0.13	3.45 ± 0.14	4.12 ± 0.16	3.98 ± 0.13	2.56 ± 0.11	4.09 ± 0.16	3.37 ± 0.13
Lys *	0.16 ± 0.01	0.23 ± 0.01	0.45 ± 0.01	0.51 ± 0.01	0.64 ± 0.02	0.38 ± 0.01	0.25 ± 0.01
Ser	4.62 ± 0.18	4.76 ± 0.17	5.32 ± 0.21	3.99 ± 0.19	4.21 ± 0.17	4.34 ± 0.16	6.04 ± 0.24
Asp	8.98 ± 0.34	9.32 ± 0.33	8.23 ± 0.32	7.98 ± 0.31	10.34 ± 0.40	8.42 ± 0.33	9.25 ± 0.37
Glu	17.65 ± 0.68	18.93 ± 0.71	18.32 ± 0.69	16.87 ± 0.64	17.34 ± 0.55	17.54 ± 0.62	16.99 ± 0.52
Thr *	3.98 ± 0.15	2.76 ± 0.11	4.12 ± 0.16	3.46 ± 0.13	5.60 ± 0.22	4.09 ± 0.16	3.78 ± 0.15
Gly	4.12 ± 0.15	5.09 ± 0.19	4.89 ± 0.17	3.98 ± 0.18	5.43 ± 0.22	4.01 ± 0.16	3.45 ± 0.13
Trp *	1.43 ± 0.06	1.21 ± 0.04	1.89 ± 0.06	0.95 ± 0.03	1.67 ± 0.06	1.45 ± 0.06	1.32 ± 0.04
Ala	4.54 ± 0.17	4.89 ± 0.16	3.98 ± 0.15	5.45 ± 0.19	4.67 ± 0.17	3.69 ± 0.14	4.56 ± 0.18
Tyr	2.57 ± 0.09	2.78 ± 0.08	3.05 ± 0.13	1.98 ± 0.07	2.23 ± 0.09	2.87 ± 0.08	3.42 ± 0.14
Pro	19.87 ± 0.87	20.12 ± 0.79	18.90 ± 0.67	19.45 ± 0.75	18.34 ± 0.78	19.67 ± 0.80	17.87 ± 0.69
Met *	0.34 ± 0.01	0.25 ± 0.01	0.43 ± 0.01	0.39 ± 0.01	0.48 ± 0.02	0.33 ± 0.01	0.46 ± 0.01
Val *	5.67 ± 0.23	4.57 ± 1.76	5.08 ± 0.21	6.12 ± 0.24	5.23 ± 0.21	5.62 ± 0.19	4.38 ± 0.17
Phe *	5.43 ± 0.21	4.78 ± 0.20	6.02 ± 0.24	5.56 ± 0.22	5.78 ± 0.19	4.76 ± 0.15	5.21 ± 0.18
Ile *	3.88 ± 0.14	4.83 ± 0.16	5.08 ± 0.21	3.98 ± 0.15	4.03 ± 0.14	4.67 ± 1.18	5.21 ± 0.21
Leu *	5.97 ± 0.24	4.65 ± 0.19	5.98 ± 0.23	6.32 ± 0.25	5.57 ± 0.22	5.90 ± 0.25	4.09 ± 0.15
T	94.65 ± 1.28	94.27 ± 2.14	98.29 ± 1.18	93.15 ± 1.19	96.32 ± 1.18	93.11 ± 1.67	91.99 ± 1.09
E	26.86 ± 0.45	23.28 ± 1.79	29.05 ± 0.48	27.29 ± 0.46	29 ± 0.45	27.2 ± 1.24	24.7 ± 0.39
N	67.79 ± 1.20	70.99 ± 1.16	69.24 ± 1.08	65.86 ± 1.10	67.32 ± 1.10	65.91 ± 1.11	67.29 ± 1.01
E/T (%)	28.38	24.70	29.56	29.30	30.11	29.21	26.85
E/NE (%)	39.62	32.79	41.96	41.44	43.08	41.27	36.71

Note: *: essential amino acids; E: total content of essential amino acids; NE: total content of non-essential amino acids; T: total content of amino acids.

**Table 5:** Information of the collected *Nitraria tangutorum* Bobr. sample in Qinghai-Tibet Plateau.

Region	Elevation (m)	Longitude	Latitude
Dagele	2679	95°45.202′	36°27.216′
Zongjia	2778	96°56.850′	36°15.959′
Nuomuhong	2703	96°28.233′	36°32.338′
Keluke Lake	2816	96°54.180′	37°19.024′
Hedong Farm	2783	96°07.799′	36°25.657′
Dulan	3198	97°59.375′	36°01.921′
Gahai	2854	97°35.766′	37°07.535′
